# What if communities held the solutions for universal health coverage?

**DOI:** 10.1186/s40249-019-0586-9

**Published:** 2019-09-05

**Authors:** John C. Reeder, Marie-Paule Kieny, Rosanna Peeling, François Bonnici

**Affiliations:** 1 Special Programme for Research and Training in Tropical Diseases (TDR), World Health Organization, Geneva, Switzerland; 2 French National Institute of Health and Medical Research, Inserm (Institut national de la santé et de la recherche médicale), Paris, France; 3 London School of Hygiene and Tropical Medicine, London, UK; 4 Bertha Centre for Social innovation and entrepreneurship, University of Cape Town, Cape Town, South Africa

**Keywords:** Social innovation, Health care delivery, Community engagement, Multidisciplinary research

## Abstract

This commentary highlights the value of community-engaged social innovations to advance health care delivery in low- and middle-income countries and to accelerate universal health coverage. It emphasizes the importance of research to guide the innovators on what works, what does not work to make their innovations sustainable and to replicate and scale them up as relevant. It also helps to demonstrate impact and to enhance uptake within the health systems.

**Electronic supplementary material:**

The online version of this article (10.1186/s40249-019-0586-9) contains supplementary material, which is available to authorized users.

## Multilingual abstracts

Please see Additional file 1 for translations of the abstract into the five official working languages of the United Nations.

## Background

Universal health coverage is one of the most pressing objectives of the World Health Organization (WHO). A billion people worldwide lack access to basic health care services, even when proven treatments exist. To extend health care and services to the most remote regions and marginalized populations, we must actively engage people and communities as the principal actors in their own health.

## Main text

Social innovation is a powerful process for this. Examples of this are diverse, and include nurse-run primary healthcare posts in rural Rwanda [1], community-led human immunodeficiency virus (HIV) testing centres in China [2], and drug shop integrated care programme in Uganda [3]. However, an essential element of innovation is that not every idea works. It is important to be bold and try new ideas, but even more important to be objective about improving or dropping them. We need to collect and analyse information about why these projects do or do not work, and to determine whether the social innovations can be scaled up, sustained and applied in different settings.

Quality research, that is reliable, ethical and answers the key questions, is necessary to make policy decisions. For this to occur, research cannot be conducted on community members as subjects; rather, research must be undertaken with community members as co-investigators. Capacity for this to occur needs to be developed to allow community members who are implementing innovations to be research generators, and for them to be able to partner with researchers in equitable collaborations.

In 2014 a collaborative undertaking emerged between the Special Programme for Research and Training in Tropical Diseases (TDR), the University of Cape Town, the University of Oxford and the London School of Hygiene and Tropical Medicine [4, 5]. The Social Innovation in Health Initiative, SIHI, was developed on the premise that solutions to many health problems can emerge from communities in low-resource settings. The initiative is dedicated to advancing the understanding and application of social innovation in the global South to address inequities in health. It has expanded in 2016 to include social innovation research hubs in China, Colombia, Malawi, Uganda and the Philippines. The country hubs provide a platform to convene the various health system actors and promote and catalyse social innovations in health through research, capacity building and advocacy. To date (July 2019), 40 social innovations in 17 countries have been showcased and studied [5, 6]. Businesses, governments, donors and civil society organizations are working together to improve healthcare access for the poor. The initiative has been developing research capacity and tools to assess whether and how these innovations strengthen health systems and reduce inequity. New global partners have joined the initiative, including the Ahimsa Fund, the Fondation Merieux, the Joint United Nations Programme on HIV/AIDS (UNAIDS), the United Nation University and the World Health Organization.

## Conclusion

*What if* people were given the opportunity to address their own health problems with locally generated solutions? *What if* these solutions were sustainable and scalable? *What if* improving healthcare also empowered people, provided economic benefit, and provided hope? Surely, this is a worthwhile topic for research, demonstrated in the thematic series on social innovation to transform health care delivery.

## Additional file

Additional file 1: Multilingual abstracts in the five official working languages of the United Nations. (PDF 344 kb)

## Data Availability

Not applicable.

## Abbreviations

HIV: Human immunodeficiency virus
TDR: Special Programme for Research and Training in Tropical Diseases, cosponsored by the United Nations Children’s Fund, the United Nations development Programme, the World Bank and the World Health Organization
UNAIDS: The Joint United Nations Programme on HIV/AIDS
