# Supplementary material for: What if communities held the solutions for universal health coverage?
Source: Infect Dis Poverty. 2019 Sep 5;8:74. doi: 10.1186/s40249-019-0586-9 (PMC6727335; doi:10.1186/s40249-019-0586-9)
Supplement: Supplementary file 1 — Multilingual abstracts in the five official working languages of the United Nations. (PDF 344 kb) [file 40249_2019_586_MOESM1_ESM.pdf]

Translation of the abstract into the five official working languages of the United Nations

ماذا لو عقدت المجتمعات حلولاً لتغطية صحية شاملة؟

جون سي ريدر، ماري بول كيني، روزانا بيلينغ وفرانسوا بونيسي.

موجز

ويبرز هذا التعليق قيمة الابتكار الاجتماعي الذي يمارسه المجتمع المحلي للنهوض بتقديم الرعاية الصحية في البلدان ذات الدخل المحدود والمتوسط والتعجيل بالتغطية الصحية العامة. ويؤكد على أهمية البحوث لتوجيه المبتكرين فيما يعمل وما لا يعمل لاستمرارية إبداعاتهم ومحاكاتها وتوسيع نطاقها حسب الاقتضاء. وأيضاً لبيان تأثيرها وتعزيز الاستيعاب داخل النظم الصحية.

Translated from English version into Arabic by Manar Kawy, Revised by Amal Alaboud, through

TRANSLATORS
WITHOUT BORDERS

社区参与社会创新推进全民健康覆盖

John C. Reeder, Marie-Paule Kieny, Rosanna Peeling and François Bonnici

摘要

本文强调了社区参与的社会创新推动了中、低收入国家提供医疗服务，加速推进全民健康覆盖进程。它强调了研究的重要性，如引导可创新的方向，促进创新的可持续发展，并将其在某些区域进行复制和扩展，还有助于发挥卫生系统的影响力，并扩大其覆盖范围。

Translated from English version into Chinese by Jin Chen

CDC CHINESE CENTER FOR DISEASE CONTROL AND PREVENTION
NATIONAL INSTITUTE OF PARASITIC DISEASES

Et si les communautés détenaient les solutions pour la couverture maladie universelle ?

John C. Reeder, Marie-Paule Kieny, Rosanna Peeling and François Bonnici

Résumé

Ce commentaire souligne la valeur de l'engagement communautaire en termes d'innovations sociales, qui ont pour objectif de faire progresser les prestations de services de santé dans les pays à faible ou moyen revenu et d'accélérer l'accès à la couverture maladie universelle. Il souligne l'importance de la recherche afin d'indiquer aux innovateurs ce qui fonctionne, ou au contraire ne fonctionne pas, dans le but de pérenniser leurs innovations et de pouvoir les reproduire et les étendre

là où elles sont pertinentes. Cela permet aussi de mesurer leur impact et d'améliorer leur intégration dans les systèmes de santé.

Translated from English version into French by Camille Onoda, Revised by Imane Bouamoud, through

TRANSLATORS WITHOUT BORDERS

Могут ли общины решить проблему всеобщего медицинского обслуживания?

Джон К. Ридер, Мари-Поль Кини, Розанна Пилинг и Франсуа Бонничи

Аннотация

В данном примечании подчеркивается значимость социальных инноваций, введённых общинами, для улучшения медицинской помощи в странах с низким/средним уровнем дохода и для ускорения всеобщего медицинского обслуживания. Здесь отмечается важность проведения исследований с целью выбора подходящего метода для создания устойчивых технологий, которые в дальнейшем будут использоваться в крупных масштабах. Исследование также продемонстрирует оказанное влияние и позволит улучшить работу систем здравоохранения.

Translated from English version into Russian by Victoria Shoptenko, Revised by Lamiya Mehtieva, through

TRANSLATORS WITHOUT BORDERS

¿Y si las comunidades tuviesen las soluciones para la Cobertura Universal de Salud?

John C. Reeder, Marie-Paule Kieny, Rosanna Peeling y François Bonnici

Resumen

El presente comentario resalta el valor de las innovaciones sociales comunitarias para impulsar la prestación de atención sanitaria en países de bajos y medianos ingresos y acelerar la cobertura universal de salud. Hace hincapié en la importancia de la investigación para guiar a los innovadores con respecto a lo que funciona y lo que no para hacer que sus innovaciones sean sostenibles y poder replicarlas y ampliarlas, según sea pertinente. Asimismo, ayuda a demostrar su impacto y a mejorar su asimilación dentro de los sistemas de salud.

Translated from English version into Spanish by Melisa Espeche, Revised by Mayra León, through

TRANSLATORS
WITHOUT BORDERS
